# Supplementary figures and images for: Alterations of the interactome of Bcl-2 proteins in breast cancer at the transcriptional, mutational and structural level
Source: PLoS Comput Biol. 2019 Dec 11;15(12):e1007485. doi: 10.1371/journal.pcbi.1007485 (PMC6927658; doi:10.1371/journal.pcbi.1007485)

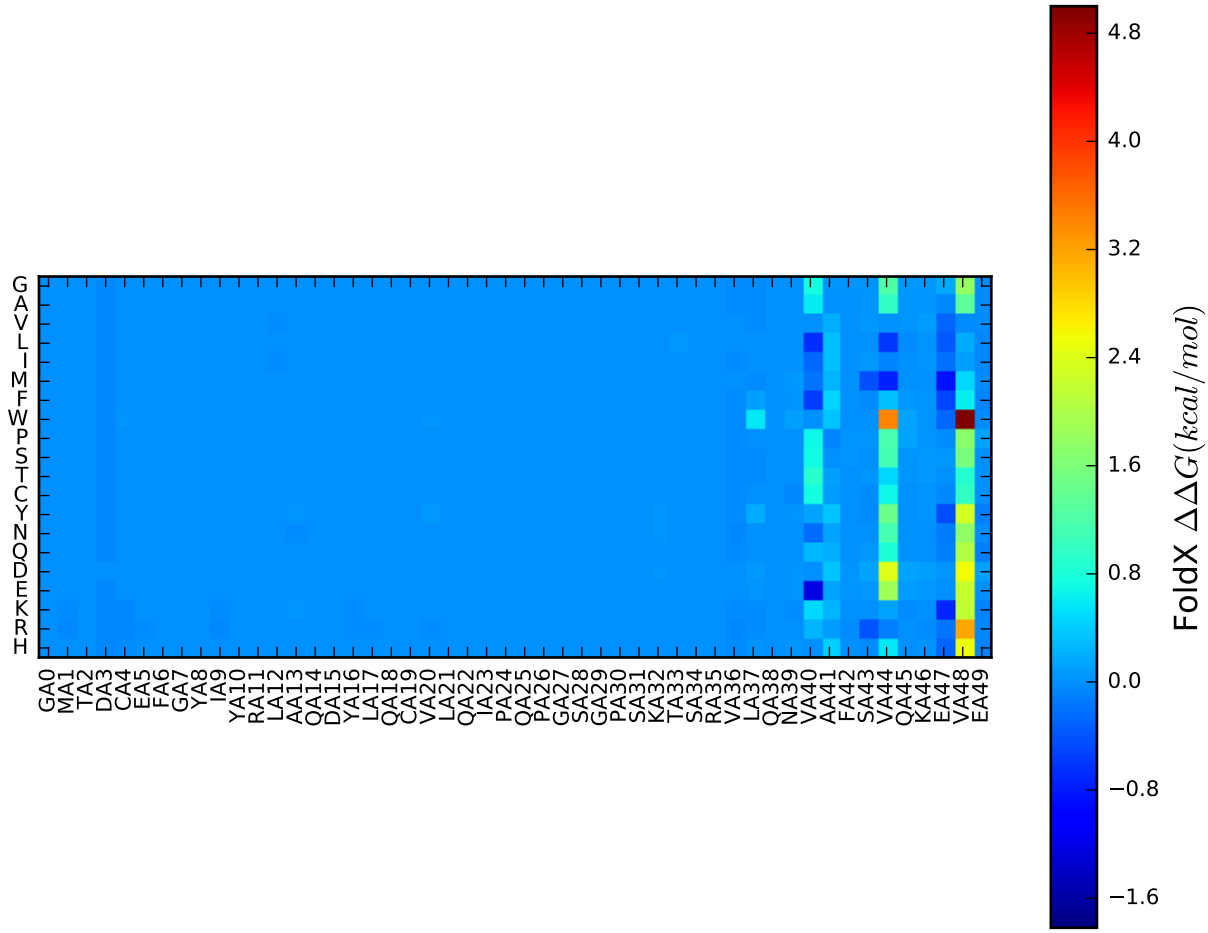

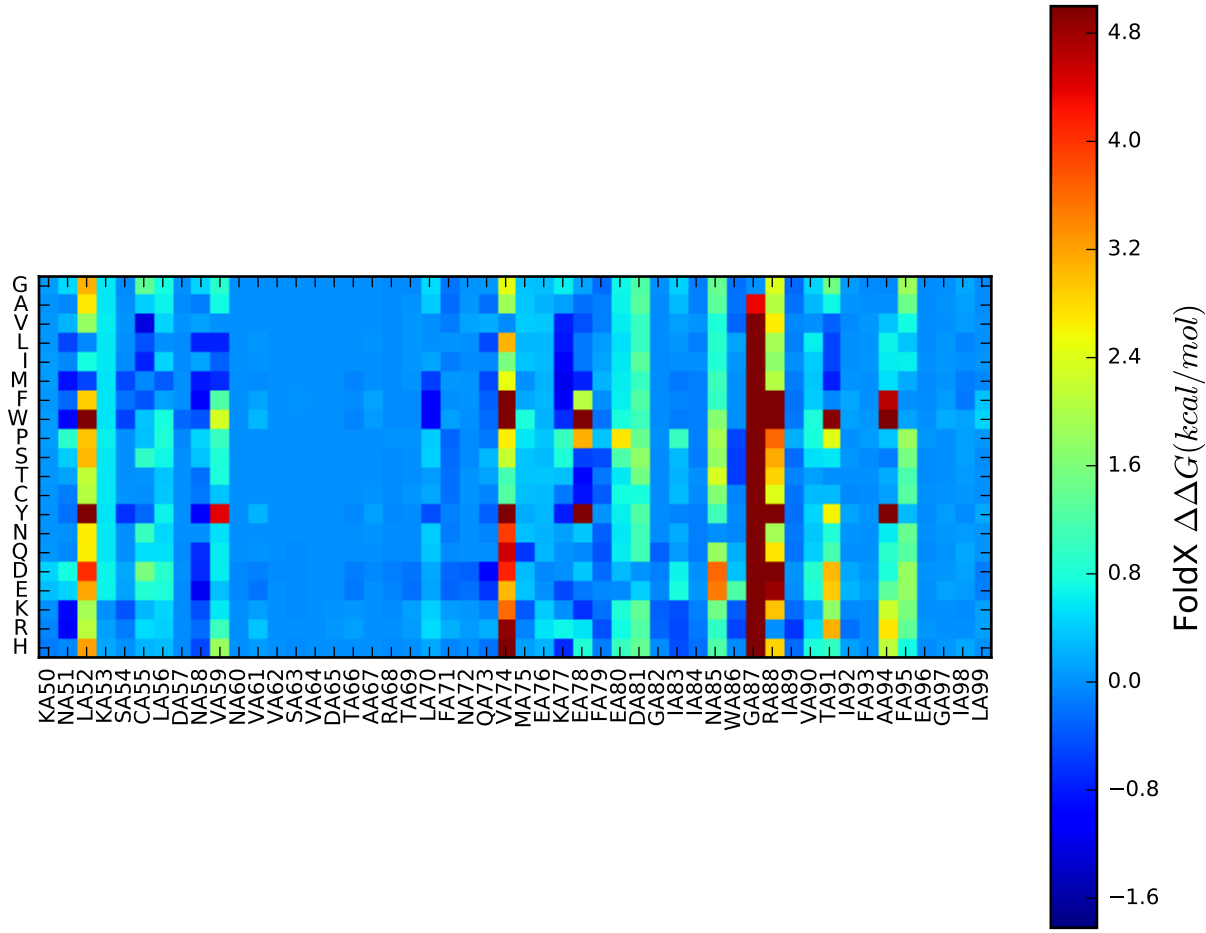

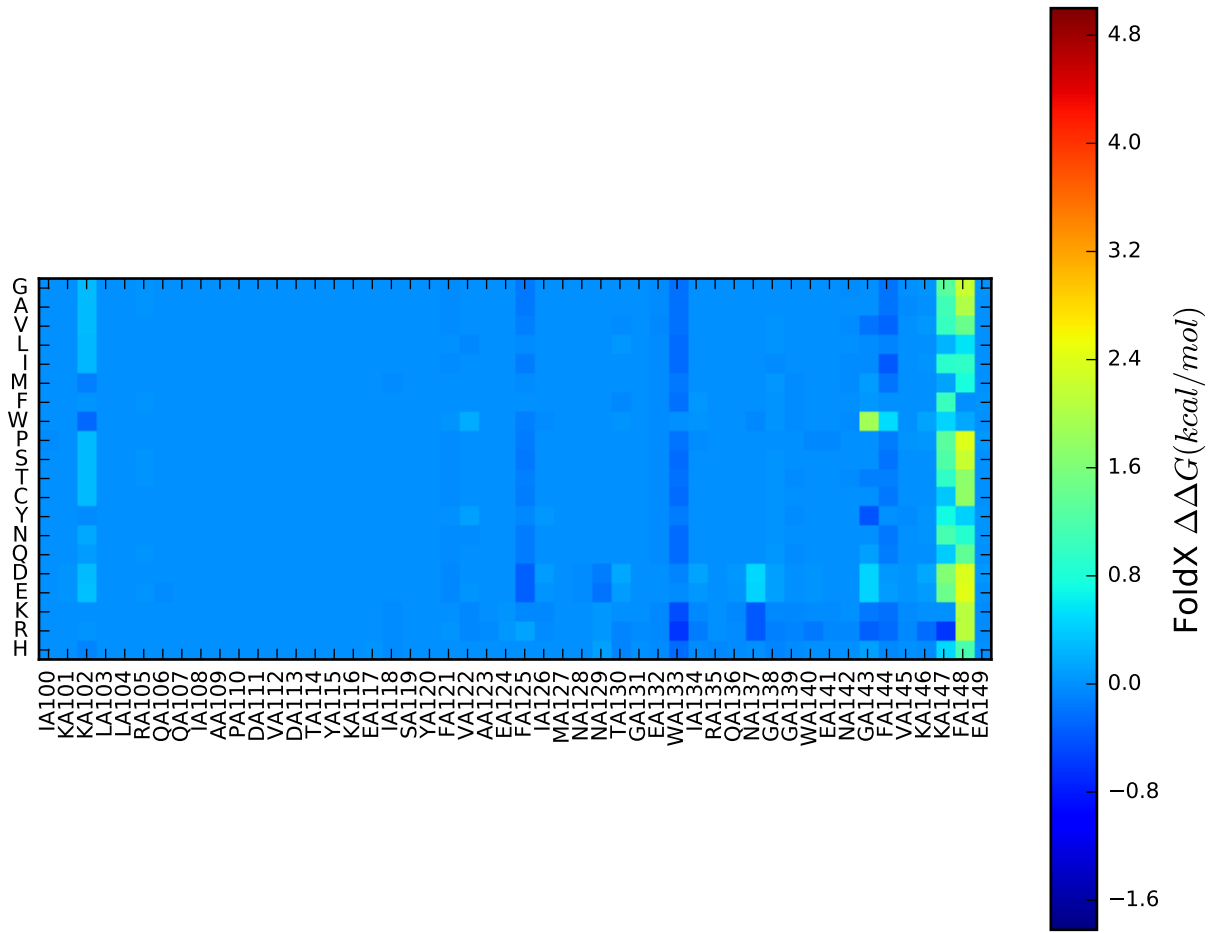

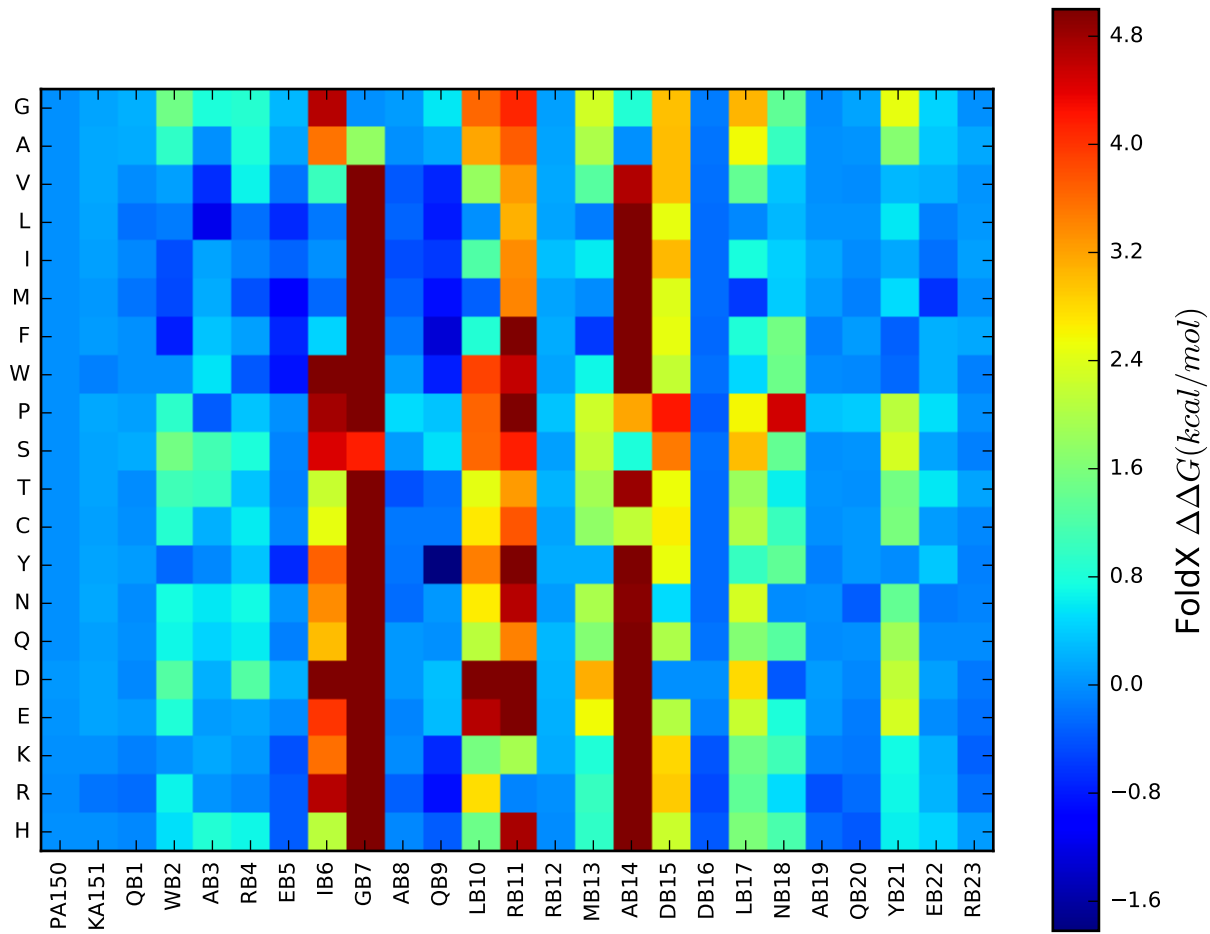

Supplement: S1 Fig — (PDF) [file pcbi.1007485.s008.pdf]
